# Impact of a *Limosilactobacillus fermentum*, Quercetin, and Resveratrol Nutraceutical on Fecal Microbiota Composition and Metabolic Activity in Healthy and Hypertensive Subjects

**DOI:** 10.3390/foods14060986

**Published:** 2025-03-14

**Authors:** Jéssica Maria Alves Brasil, Nathalia Caroline de Oliveira Melo, Karoliny Brito Sampaio, Paulo César Trindade da Costa, Hatice Duman, Sercan Karav, Marcos dos Santos Lima, Evandro Leite de Souza, José Luiz de Brito Alves

**Affiliations:** 1 Department of Nutrition, Health Sciences Center, Federal University of Paraíba, João Pessoa 58051-900, PB, Brazil; jessik_brasil@hotmail.com (J.M.A.B.); nathalia.melo@ufpe.br (N.C.d.O.M.); karolbsampaio@gmail.com (K.B.S.); paulocesarnutricionista@gmail.com (P.C.T.d.C.); els@academico.ufpb.br (E.L.d.S.); 2 Department of Molecular Biology and Genetics, Çanakkale Onsekiz Mart University, Çanakkale 17000, Türkiye; hatice.duman@comu.edu.tr (H.D.); sercankarav@comu.edu.tr (S.K.); 3 Department of Technology, Federal Institute of Sertão de Pernambuco, Petrolina 56300-000, PE, Brazil; marcos.santos@ifsertao-pe.edu.br

**Keywords:** bioactive compounds, intestinal microbiota, hypertension, prebiotic, probiotic

## Abstract

A promising strategy to improve the gut microbiome in hypertension is to target the gut microbiota. This study evaluated the effects of a potential nutraceutical product composed of three strains of *Limosilactobacillus (L.) fermentum*, quercetin, and resveratrol on the intestinal microbiome of healthy and hypertensive subjects. The nutraceutical product consisting of strains of *L. fermentum* 139, 263 and 296, fructooligosaccharides (200 mg), quercetin (160 mg), and resveratrol (150 mg) (LfQR) was added to the in vitro fecal fermentation process occurring for 48 h. Fecal samples of healthy and hypertensive subjects were allocated into four groups: (i) healthy controls (CTL); (ii) healthy controls with the addition of LfQR (CTL + LfQR); (iii) hypertensive (HTN) subjects; and (iv) hypertensive subjects with the addition of LfQR (HTN + LfQR). The diversity and composition of the fecal microbiota and the production of microbial metabolites were evaluated. CTL and HTN groups exhibited a distinct gut microbiota composition, as shown by the β-diversity assessment. The addition of the potentially nutraceutical-modulated β-diversity was similar between CTL and HTN groups, suggesting a similar gut microbiome composition after nutraceutical addition. The addition of the nutraceutical product increased the relative abundance of Enterobacteriaceae in the CTL group and that of Lachnospiraceae in the HTN group. The nutraceutical media showed higher levels of sugars (maltose, fructose, and glucose), lactic acid, ethanol, succinic acid, and acetic acid compared to the CTL and HTN media. Although the results are heterogeneous between healthy and hypertensive fermentation media, it was demonstrated that the nutraceutical formulation can modulate the intestinal microbiota and its metabolic activity.

## 1. Introduction

Arterial hypertension is a public health problem with a complex and multifactorial etiology [1], and evidence suggests a bidirectional relationship between the onset and maintenance of hypertension and gut dysbiosis [2]. Intestinal dysbiosis is characterized as a qualitative and/or quantitative change in the composition of the intestinal microbiota, both at a taxonomic and functional level. Intestinal dysbiosis can be triggered by endogenous and exogenous factors, such as the host’s physiology and immune system, health-damaging dietary patterns, recurrent antibiotic use, and/or pre-existing comorbidities [3].

High blood pressure can affect the structure and function of the gut and increase gut permeability, favoring or exacerbating intestinal dysbiosis, which facilitates the translocation of microbial metabolites into the bloodstream and activates inflammatory processes. It is recognized that inflammation promotes the sympathetic hyperactivity, elevation and maintenance of blood pressure [4]. Adjuvant therapeutic strategies related to intestinal microbiota modulation have been proposed for preventing and treating arterial hypertension [5], and dietary management and supplementation with prebiotics, probiotics, or synbiotics are often indicated [6]. Positive modulation of the gut microbiota may result in beneficial effects on blood pressure through various mechanisms, including reduced systemic inflammation and the improvement of cardiac autonomic function [7,8].

Prebiotics are dietary compounds that are selectively used by the intestinal microbiota to provide health benefits to the host [9]. Although prebiotic function is mainly exerted by substances derived from indigestible carbohydrates, bioactive molecules present in plant foods, such as phenolic compounds, also exert a prebiotic function [10,11]. Phenolic compounds, including quercetin and resveratrol, have well-established functional properties, including anti-inflammatory, antioxidant, and cardioprotective effects [12,13,14]. In addition, phenolic compounds have been shown to be effective in modulating the intestinal microbiota, stimulating the growth of beneficial bacteria and reducing pathogenic bacteria [15].

Probiotics are live microorganisms that provide health benefits to the host when administered in adequate doses. These microorganisms are responsible for modulating the intestinal microbiota and exerting anti-inflammatory and protective effects on the intestinal epithelium [16,17,18], with evidence that the interactions between phenolic compounds and probiotics may enhance their beneficial effects on host health [19,20,21].

Considering mutually beneficial interactions, the ingestion of both probiotics and phenolic compounds in potentially symbiotic products could be an option with which to improve and extend their functionalities targeting the intestinal microbiota [22]. Recently, a nutraceutical formulated with probiotic strains of *Limosilactobacillus fermentum* 139, 263, and 296, quercetin and resveratrol (LfQR) with proven antioxidant properties has been developed [23], but its effect on the intestinal microbiota of healthy and hypertensive adults has not yet been elucidated.

This study aimed to evaluate the effects of this novel nutraceutical on the composition, diversity, and metabolic activity of the fecal microbiota of healthy and hypertensive adults during an in vitro fecal fermentation. The hypothesis tested is that a nutraceutical consisting of a mix of *L. fermentum* strains, quercetin, and resveratrol would induce beneficial changes in the composition, diversity, and metabolic activity of the fecal microbiota of healthy and hypertensive adults.

## 2. Methods

### 2.1. Microorganisms

*L. fermentum* 139, 263, and 296 strains were used for this study. To obtain the cell suspensions, the strains were maintained individually under anaerobiosis (Anaerobic System Anaerogen, Oxoid, Hampshire, UK) and cultured in MRS broth (Mann, Rogosa and Sharpe, HiMedia, Mumbai, India) at 37 °C for 20–24 h until they reached the stationary growth phase. The cell cultures were harvested (4000× *g*, 10 min, 4 °C), washed with sterile saline (0.85 g/100 mL NaCl), and resuspended in sterile distilled water. The cell suspensions had viable cell counts of approximately 10 log CFU/mL when plated on MRS agar (HiMedia), and a mix of suspended cells in a 1:1:1 ratio was obtained.

### 2.2. Preparation of Nutraceutical with L. Fermentum, Quercetin, and Resveratrol

For the formulation of the nutraceutical, a mixed suspension of *L. fermentum* strains 139, 263, and 296, sterile distilled water (1 mL), and fructooligosaccharides (200 mg) [FOS, 20% *w*/*v*], a well-known cryoprotectant for bacterial cells, was used. The mixture was frozen at −80 °C for 24 h and lyophilized (temperature −55 ± 2 °C, vacuum pressure < 138 μHG, lyophilization rate 1 mm/h) for approximately 40 h using a bench-top lyophilizer (Liotop^®^, Model L-101, São Carlos, SP, Brazil).

Quercetin (160 mg; Sigma-Aldrich, St. Louis, MI, USA) and resveratrol (150 mg; Sigma-Aldrich) were added to the powder. The mixture was homogenized and transferred to sterile amber bottles with screw caps, placed in desiccant with silica gel, and stored at 4 ± 0.5 °C with controlled relative humidity until use [24]. The nutraceutical was rehydrated with sterile distilled water (10 mL, 25 ± 0.5 °C) for 15 min and subjected to simulated gastrointestinal digestion.

### 2.3. Simulated Gastrointestinal Digestion of the Nutraceutical

Continuous chemical processes and mechanical stirring were carried out in an incubator (37 ± 0.5 °C) to simulate the conditions and peristalsis movements of the mouth, stomach, duodenum, and ileum. For the stomach condition (exposure time 120 min, 37 ± 0.5 °C, 130 rpm), the pH was adjusted to 2.0 with 1 M HCl, and a solution of pepsin (25 mg/mL) in 0.1 M HCl was added (0.05 mL/mL,). For the duodenum conditions (exposure time 30 min, 37 ± 0.5 °C, 45 rpm), the pH was increased to 5 with NaHCO_3_ 0.1 M, and a solution with 12 g of bovine bile salt/L was added. For ileal conditions (exposure time 60 min, 37 ± 0.5 °C, 45 rpm), the pH was increased to 6.5 with 0.1 M NaHCO_3_ for 60 min [24]. Enzymes, bile salts, and reagents were purchased from Sigma-Aldrich.

### 2.4. Preparation of Human Fecal Inoculum

The experimental procedures were approved by an Institutional Committee for Ethics in Human Research (Federal University of Paraíba, João Pessoa, PB, Brazil) under protocol 5.626.691 and 5.049.860, and followed the guidelines of the National Health Council (Resolution 466, 2012).

Fecal samples were donated by six healthy adults (the control group, CTL, consisting of three men and three women over 18 years old) and six hypertensive adults (the hypertension group, HTN, consisting of three men and three women over 18 years old). The samples were collected from healthy donors who reported no medical conditions and from hypertensive donors diagnosed with the disease for at least one year with no other comorbidity. The selected healthy and hypertensive donors had not taken any prebiotic or probiotic supplements in the six months before stool collection, and had not taken any antibiotics or other medications that affect the gut microbiota composition, except for antihypertensive medications commonly used in hypertensive individuals (Table 1).

Fecal samples were deposited in sterile flasks using an anaerobic generator system (AnaeroGen, Thermo Fisher, Waltham, MA, USA). For each deposited sample, 20 g of fresh feces was homogenized, mixed in equal proportions (1:1:1:1:1:1:1, *w*/*w*), diluted (1:10, *w*/*v*) in sterile phosphate-buffered saline (PBS; 0.1 mol/L; pH 7.4), homogenized by stirring (2 min, 200 rpm), and filtered with a sterile triple-layer gauze to remove larger particles.

### 2.5. In Vitro Fecal Fermentation of the Nutraceutical

The fecal fermentation system was designed according to an early study [24] and following the separation of the healthy group from the hypertensive group, comprising four different groups: a medium composed of 50% (*v*/*v*) of the fecal inoculum of healthy subjects and 50% (*v*/*v*) of a sterile fermentation medium (control group, CTL); a medium with a nutraceutical composed of 40% (*v*/*v*) of the fecal inoculum of healthy subjects, 40% of (*v*/*v*) a sterile fermentation medium, and 20% of a pre-digested nutraceutical (*w*/*v*) (CTL + LfQR group); a medium composed of 50% (*v*/*v*) of the fecal inoculum of hypertensive subjects and 50% (*v*/*v*) of a sterile fermentation medium (HTN group); and a medium with a nutraceutical composed of 40% (*v*/*v*) of the fecal inoculum of hypertensive subjects, 40% (*v*/*v*) of a sterile fermentation medium, and 20% of a predigested nutraceutical (HTN + LfQR group).

The sterile fermentation medium used in the fecal fermentation was formulated with 4.5 g NaCl, 4.5 g KCl, 1.5 g NaHCO_3_, 0.69 g MgSO_4_, 0.8 g L-cysteine, 0.5 g KH_2_PO_4_, 0.5 g K_2_HPO_4_, 0.4 g bile salt, 0.08 g CaCl_2_, 0.005 g FeSO_4_, 1 mL of Tween 80, and 4 mL of resazurin solution (0.025%, *v*/*v*) as an anaerobic indicator, diluted in 1 L of distilled water and sterilized in an autoclave (121 °C, 1 atm, 15 min). Aliquots were collected after 48 h of fecal fermentation and centrifuged (4000× *g*, 10 min, 4 °C), the supernatants were used to measure the contents of organic acids, and the pellets were used for metagenomic analysis.

### 2.6. Identification and Quantification of Bacterial Groups During Fecal Fermentation

A 0.5 mL aliquot of each fecal fermentation medium was collected for the identification and quantification of bacterial groups using the 16S RNA sequencing technique. The bacterial diversity was assessed via the high-throughput sequencing of the 16S rRNA V3/V4 region, employing 341F (CCTACGGGRSGCAGCAG) and 806R (GGACTACHVGGGTWTCTAAT) primers. The 16S rRNA libraries were sequenced with the MiSeq sequencing system (Illumina Inc., San Diego, CA, USA) using the standard Illumina primers provided in the kit, in 300 cycles (paired-end sequencing with 200 bp). Libraries were prepared and sequenced by Neoprospecta (Florianópolis, SC, Brazil).

After sequencing, we removed truncated and low-quality reads from the data (Phred score < 20) using the Trimmomatic tool [25]. Paired sense and antisense sequence reads were merged into contigs, and singletons and chimeras were removed. The sequence files were imported into QIIME2 version 2021 and processed for the removal of noisy, chimeric sequences and singletons, the joining of paired-end reads, dereplication, and the obtainment of amplicon sequence variants (ASVs) using the DADA2 plugin.

A phylogenetic tree was constructed using QIIME2’s fragment insertion SEPP plugin with the Silva reference database [25], and the taxonomic composition was assessed with QIIME2’s q2-feature-classifier plugin. QIIME2 resulting files were imported into R using the phyloseq package. The sequences are registered in NCBI and identified as PRJNA1221916.

### 2.7. Evaluation of the Metabolic Activity of the Intestinal Microbiota

After fermentation, 1.5 mL aliquots of each medium were used for the determination of the contents of sugars, lactic acid, and short-chain fatty acids with the high-performance liquid chromatography technique (HPLC) using the chromatograph model 1260 Infinity LC (Agilent Technologies, St. Clara, CA, USA) with a quaternary solvent pump (model G1311C), a thermostatic column (model G1316A), an automatic degasser and an autosampler (model G1329B), coupled to a diode array detector (DAD) (model G1315D) and a refractive index detector (RID) (model G1362A). The following analytical conditions were used: an Agilent Hi-Plex H column (7.7 300 mm, 8 μ), mobile phase 0.004 mol/L H_2_SO_4_ in ultrapure water, and flow rate 0.7 mL/min. Data were processed using OpenLAB CDS ChemStation Edition software (Agilent, St. Clara, CA, USA, version LTS 01.11). The peaks of the HPLC samples obtained in the analysis were identified by comparing their retention times with those of the sugar and organic acid patterns (Sigma Aldrich, St. Louis, MA, USA), and the average areas of the peaks were used for quantification.

### 2.8. Statistical Analysis

All tests were performed in triplicate on three different occasions, and the results were expressed as mean ± standard deviation. The normality of the data was evaluated by the Shapiro–Wilk test and the one-way ANOVA test, followed by the Bonferroni post-test. A *p*-value < 0.05 was considered statistically significant. The statistical analysis was performed using the software STATA program (v15.0, College Station, TX, USA). Intestinal microbiota analysis was performed using R software (Version 4.3.3), an taxa bar plots and diversity analyses were performed using the microViz R package. Alpha diversity was calculated as the Shannon index, and the beta diversity metric consisted of Bray–Curtis/unweighted UniFrac distance. Differential analysis was conducted with the R package microbiomeMarker using the ANOVA-like differential expression tool (ALDEx2) and linear discriminant analysis effect size (LEfSe).

## 3. Results

**Figure 1:**
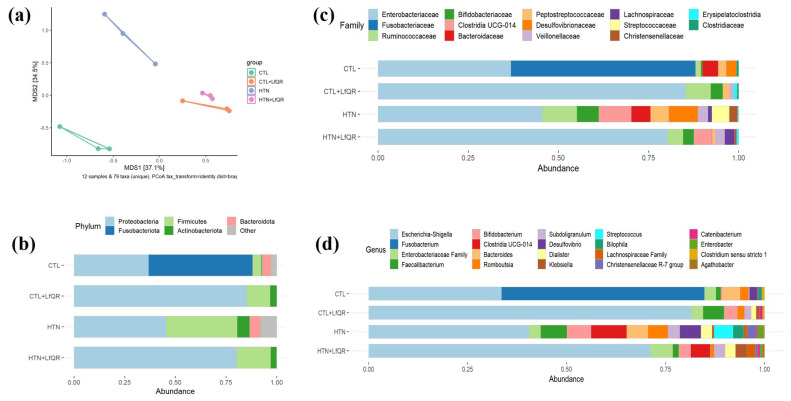
Beta diversity and relative abundance of fecal microbiota after 48 h of in vitro fecal fermentation. (**a**) Beta diversity of fecal microbiota after 48 h of in vitro fecal fermentation; (**b**) Phylum relative abundance of fecal microbiota after 48 h of in vitro fecal fermentation; (**c**) Family relative abundance of fecal microbiota after 48 h of in vitro fecal fermentation; (**d**) Genus relative abundance of fecal microbiota after 48 h of in vitro fecal fermentation.

The general characteristics of the healthy and hypertensive donors of the fecal samples are shown in Table 1. The β-diversity of fecal microbiota differed between CTL and HTN groups, as shown by the Bray–Curtis dissimilarity ratio and weighted UniFrac distances (Figure 1a), indicating a distinct gut microbiota composition. The nutraceutical formulated with *L. fermentum*, quercetin, and resveratrol modulated the β-diversity similarly in CTL and HTN groups after fecal fermentation (Figure 1a).

The α-diversity did not differ between the CTL and HTN groups (Supplementary Figure S1). According to ALDEX2, a method of differential abundance analysis, the CTL group had a reduced relative abundance of Firmicutes and an increased relative abundance of Fusobacteriota at the phylum level after fecal fermentation. In contrast, the HTN group had an increased relative abundance of Firmicutes after fecal fermentation (Figure 1b; Supplementary Figure S2). The nutraceutical reduced the relative abundance of the phylum Fusobacteriota and increased the relative abundance of the phylum Proteobacteria in CTL and HTN groups after fecal fermentation (Figure 1b; Supplementary Figure S2).

The nutraceutical increased the relative abundance of the Enterobacteriaceae and Lachnospiraceae families in both the CTL group (CTL versus CTL + LfQR) and HTN group (HTN versus HTN + LfQR) after fecal fermentation, respectively (Figure 1c). The nutraceutical also increased the relative abundance of *Escherichia-Shigella* genera in both the CTL and HTN groups (Figure 1d).

LDA analysis showed an enrichment of Lachnospiraceae and Lachnospirales in the HTN + LfQR group (HTN versus HTN + LfQR), as well as of Proteobacteria, Gammaproteobacteria, Enterobacteriales, and *Escherichia-Shigella* in the CTL + LfQR group (CTL versus CTL + LfQR) (Supplementary Figure S3).

The contents of maltose, glucose, fructose, lactic acid, acetic acid, and succinic acid were higher in the HTN + LfQR and CTL + LfQR groups compared to the CTL and HTN groups after fecal fermentation (*p* ≤ 0.05), while the contents of rhamnose were lower in the HTN and CTL groups compared to the HTN + LfQR and CTL + LfQR groups (*p* ≤ 0.05). In addition, the HTN + LfQR group had a lower content of propionic acid compared to the HTN group after fecal fermentation (*p* ≤ 0.05) (Table 2).

## 4. Discussion

The results of this study showed that a novel nutraceutical formulated with *L. fermentum* strains, quercetin, and resveratrol promoted changes in composition, diversity, and microbial metabolic activity during in vitro fermentation with fecal inoculum from healthy and hypertensive subjects. It was demonstrated that the *L. fermentum* strains used to formulate the tested nutraceutical have probiotic properties linked to the modulation of the gut microbiota and improvements in metabolic parameters, in addition to antibacterial effects [21]. In addition, quercetin and resveratrol can modulate the gut microbiota and reduce the severity of cardiometabolic diseases [26].

The nutraceutical formulation consists of probiotics and prebiotics. Probiotics can compete with other microorganisms in the gut microbiota, especially pathogens, to improve gut health [27]. Prebiotics can be metabolized by bacteria, such as the probiotics in the nutraceutical itself, to produce metabolites that are important for health, such as SCFA. In addition, prebiotics such as quercetin and resveratrol have important antioxidant and anti-inflammatory activities, thus also contributing to barrier function and overall intestinal function [26].

The alpha diversity of fecal microbiota after 48 h of fermentation in CTL + LfQR and HTN + LfQR groups was lower than that in CTL and HTN groups, while the fecal media with the nutraceutical had a similar beta diversity after fecal fermentation. Although the nutraceutical did not increase the richness of taxons in HTN, an increase in the relative abundance of the order of the Lachnospiraceae family and Lachnospirales was observed in the HTN group. A previous study showed that the administration of *L. fermentum* 139, 263, and 296 strains for four weeks reduced alpha diversity and increased the relative abundance of the Lachnospiraceae family in the gut microbiota of rats fed a high-fat diet [28], suggesting a possible pattern of gut microbiota modulation linked to these *L. fermentum* strains.

The increased diversity and richness of bacterial species present in the gut microbiota have been proposed as an important marker of gut health, as it is associated with conditions of gut stability and the better performance of different microbial functions, benefiting the local and systemic host health [29]. The enrichment of Lachnospiraceae in the fecal microbiota of the HTN + LfQR group highlights the need for further studies to understand whether or not the significant reduction in the diversity of different bacterial groups, to the detriment of the increase in the abundance of this family, is a beneficial mechanism promoted by the *L. fermentum* strains used to formulate the tested nutraceutical.

The family Lachnospiraceae colonizes the intestinal lumen from birth, and its abundance and richness increase during the host life, depending on its diet and lifestyle. Among the 80 genera of Lachnospiraceae family, some are major producers of short-chain fatty acids (SCFAs) (ex., *Blautia* and *Roseburia*), while others, such as *Ruminococcus*, in high abundance, are associated with various intra- and extraintestinal diseases, such as Chron’s disease, obesity, and glucose metabolism disorders; this family therefore has controversial effects on host health [30].

The presence of the nutraceutical in fermentation media containing fecal inoculum from healthy and hypertensive individuals increased the relative abundance of the Proteobacteria phylum and one of its bacterial families, Enterobactericeae. Proteobacteria are classified as Gram-negative and lipopolysaccharide (LPS)-producing bacteria, with evidence of their association with an increased risk of gut microbiota dysbiosis [31]. Although Enterobactericeae are present in the human intestinal tract and are a normal part of the gut microbiota, a cohort showed that an increased relative abundance of Enterobactericeae was directly associated with increased blood pressure and inflammatory markers in adults with obesity and cardiovascular disease [32]. However, whether the increase in Proteobacteria and Enterobactericeae abundance promoted by the tested nutraceutical could promote health damage remains to be investigated.

At the genus level, *Escherichia-Shigella*, which also belongs to the phylum Proteobacteria and the family Enterobactericeae, was abundant in the CTL + LfQR and HTN + LfQR groups after fecal fermentation. These genera could play an important role in hypertensive conditions [33]. The increase in bacteria belonging to the family Enterobacteriaceae in the healthy group may increase the likelihood of developing hypertension, as *Escherichia-Shigella* can degrade choline into trimethylamine-N-oxide (TMAO), a metabolite associated with increased cardiovascular risk due to its induction of an inflammatory response, oxidative stress, and vascular endothelial damage [34,35].

In contrast, the HTN + LfQR group showed a higher relative abundance of some beneficial bacterial groups after fecal fermentation, such as the Lachnospiraceae family, while the CTL + LfQR group showed an increase in the relative abundance of the genus *Feacalibacterium*. The bacterial species forming this family and genus are fermentative microorganisms capable of producing SCFAs due to dietary fiber fermentation [36]. Results from a cohort showed a negative correlation between Lachnospiraceae fecal abundance and blood pressure, and a positive correlation between Lachnospiraceae fecal abundance and intestinal butyrate contents [37].

Furthermore, an increase in Lachnospiraceae abundance was observed in the gut microbiota of adults after they adopted a healthy dietary pattern, and this increase was positively associated with a reduction in systolic and diastolic blood pressure [38]. One of the early studies showed that treatment with quercetin and resveratrol in rats fed a high-fat diet decreased the abundance of the Lachnospiraceae family [26]. It suggested that *L. fermentum* strains may have a beneficial effect on the abundance of Lachnospiraceae when combined with quercetin and resveratrol.

Increased abundance of *Feacalibacterium* in the gut microbiota is associated with improved renal function and decreased inflammation in the kidneys of rats with chronic kidney disease [39]. Although the impacts of the intestinal abundance of *Feacalibacterium* on blood pressure regulation have not been clarified, the increased abundance of this bacterial genus may be beneficial for preventing a hypertensive pathology due to the close relationship between renal function and blood pressure control.

The increase in the relative abundance of the genus *Bifidobacterium*, as observed in the CTL + LfQR group after fecal fermentation, was associated with antihypertensive effects. Administration of *Bifidobacterium breve* reduced blood pressure in spontaneously hypertensive rats [40]. Similarly, supplementation with *Bifidobacterium longum* to obese adult mice promoted increased expression of angiotensin-converting enzyme 2, supporting its potential beneficial effects on blood pressure control [41].

In general, the microbiota diversity of individuals with hypertension is more compromised than its abundance. The increased abundance of the phylum Firmicutes, genera *Megasphaera*, *Escherichia*_*Shigella*, and *Klebsiella*, and the decreased abundance of the phylum Bacteroidetes, genera *Bifidobacterium*, *Faecalibacterium*, *Roseburia*, and *Ruminococcus* may be associated with hypertension. Furthermore, intestinal microbial metabolism in hypertension seems to be more involved in lipopolysaccharide biosynthesis, membrane transport and steroid degradation [42].

When evaluating the effect of the nutraceutical on metabolic activity in the fecal microbiota, it was observed that the CTL + LfQR and HTN + LfQR groups had higher contents of sugars, such as fructose, glucose, and maltose, than the CTL and HTN groups, respectively. In addition, there was an increase in the contents of lactic acid in the CTL + LfQR and HTN + LfQR groups, which could be linked to the capability of *L. fermentum* to cause lactifrom carbohydrate metabolism [43]. Lactic acid-producing bacteria stimulate enterocyte proliferation and maintain intestinal epithelial integrity [44]; they also suppress inflammation by modulating the gut microbiota [45].

When the production of SCFAs (acetate, propionate, and butyrate) was examined, the CTL + LfQR and HTN + LfQR groups had higher contents of acetic acid compared to propionic and butyric acid, especially when compared to the CTL and HTN groups. Even though butyric acid was not quantified in some of the fermentation media, the ratio of butiril-CoA: acetate-CoA-transferase is an important pathway for butyric acid biosynthesis. Therefore, although butyric acid was not observed, the presence of the tested nutraceutical could favor an increase in the concentration of its precursor [46,47]. In general, SCFAs are microbial metabolites associated with host health benefits, mainly due to their epigenetic modulation of anti-inflammatory pathways and the immune system [46,47].

## 5. Conclusions

The proposed nutraceutical, formulated with a mix of *L. fermentum* 139, 263 and 296, quercetin, and resveratrol, was effective in modifying the composition and parameters linked to the microbial metabolic activity of the fecal microbiota of healthy and hypertensive subjects during in vitro fermentation. One of the major limitations of this study is that it did not track dietary patterns and other lifestyle factors that may have affected the fecal sample donors. Experimental studies and clinical trials can be conducted to obtain results that will improve our understanding of the effects of this nutraceutical formulation.

## Figures and Tables

**Table 1:** General characteristics of healthy and hypertensive donors of fecal samples.

	Donor	Sex	Age (Years)	Antibiotic Use (Last Six Months)	Probiotic/Prebiotic/Symbiotic Use (Last Six Months)	Number of Antihypertensive (Daily Use)
	1	Male	37	No	No	-
Healthy donors	2	Male	29	No	No	-
	3	Male	28	No	No	-
	4	Female	28	No	No	-
	5	Female	42	No	No	-
	6	Female	25	No	No	-
	7	Male	37	No	No	1
Hypertensive donors	8	Male	36	No	No	2
	9	Male	42	No	No	2
	10	Female	54	No	No	1
	11	Female	53	No	No	1
	12	Female	47	No	No	1

**Table 2:** Contents of sugar and organic acids in the medium with and without a nutraceutical formulated with *L. fermentum*, quercetin, and resveratrol after 48 h of in vitro fecal fermentation with healthy and hypertensive individuals.

Paremeters	CTL	CTL + LfQR	HTN	HTN + LfQR	F Value	*p*-Value
Sugars						
Maltose	0.00 ± 0.00	0.93 ± 0.00 ^#α^	0.00 ± 0.00	0.66 ± 0.03 *	2228.25	<0.001
Glucose	0.00 ± 0.00	0.75 ± 0.00 ^#α^	0.00 ± 0.00	0.54 ± 0.05 *	722.52	<0.001
Fructose	0.04 ± 0.03	3.41 ± 0.02 ^#α^	0.00 ± 0.00	3.65 ± 0.03 *	17,264.83	<0.001
Rhaminose	0.11 ± 0.05	0.00 ± 0.00 ^#^	0.09 ± 0.02	0.00 ± 0.00 *	11.32	<0.001
Organic acids						
Lactic acid	0.00 ± 0.00	1.70 ± 0.01 ^#α^	0.00 ± 0.00	1.75 ± 0.02 *	35,593.64	<0.001
Acetic acid	0.32 ± 0.01 *	0.65 ± 0.00 ^#α^	0.37 ± 0.01	0.67 ± 0.00 *	2371.93	<0.001
Propionic acid	0.05 ± 0.09 *	0.04 ± 0.08	0.45 ± 0.05	0.21 ± 0.00 *	26.49	<0.001
Butyric acid	0.00 ± 0.00	0.00 ± 0.00	0.06 ± 0.10	0.00 ± 0.00	1.00	0.44

HTN: hypertensive fermentation medium. HTN-LfQR: hypertensive fermentation medium with LfQR. CTL: control fermentation medium; CTL + LfQR: control fermentation medium with LfQR. LfQR: nutraceutical formulated with *L. fermentum*, quercetin, and resveratrol. Data are expressed as average ± standard deviation. *p*-values are based on the one-way ANOVA test followed by the Bonferroni post-test. * vs. HTN, ^#^ vs. CTL, ^α^ vs. HTN + LfQR.

## Data Availability

The original contributions presented in the study are included in the article/Supplementary Material, further inquiries can be directed to the corresponding author.

## Supplementary Materials

The following supporting information can be downloaded at: https://www.mdpi.com/article/10.3390/foods14060986/s1, Figure S1: Microbial alpha diversity in media with and without a nutraceutical formulated with *L. fermentum*, quercetin, and resveratrol after 48 h of in vitro fecal fermentation with healthy and hypertensive individuals; Figure S2: Relative abundance of microbial phyla in vitro colonic fermentation; Figure S3: LDA analysis of microbial genera in vitro colonic fermentation.
