# Supplementary material for: Impact of a Limosilactobacillus fermentum, Quercetin, and Resveratrol Nutraceutical on Fecal Microbiota Composition and Metabolic Activity in Healthy and Hypertensive Subjects
Source: Foods. 2025 Mar 14;14(6):986. doi: 10.3390/foods14060986 (PMC11941034; doi:10.3390/foods14060986)
Supplement: Supplementary file 1 [file foods-14-00986-s001.zip › foods-3498698-supplementary.pdf]

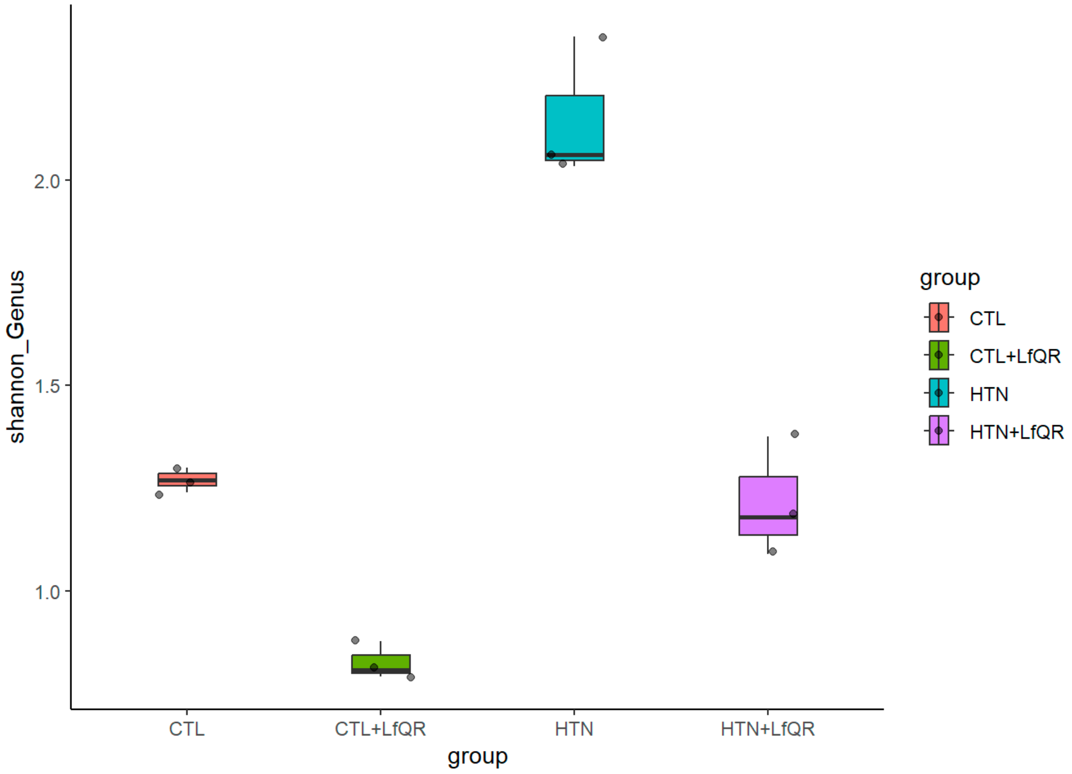

Supplementary Figure S1. Microbial alpha diversity in media with and without a nutraceutical formulated with *L. fermentum*, quercetin, and resveratrol after 48 h of *in vitro* fecal fermentation with healthy and hypertensive individuals.

HTN: Hypertensive fermentation medium. HTN-LfQR: Hypertensive fermentation medium with LfQR. CTL: Control fermentation medium; CTL+ LfQR: Control fermentation medium with LfQR. LfQR: Nutraceutical formulated with *L. fermentum*, quercetin and resveratrol.

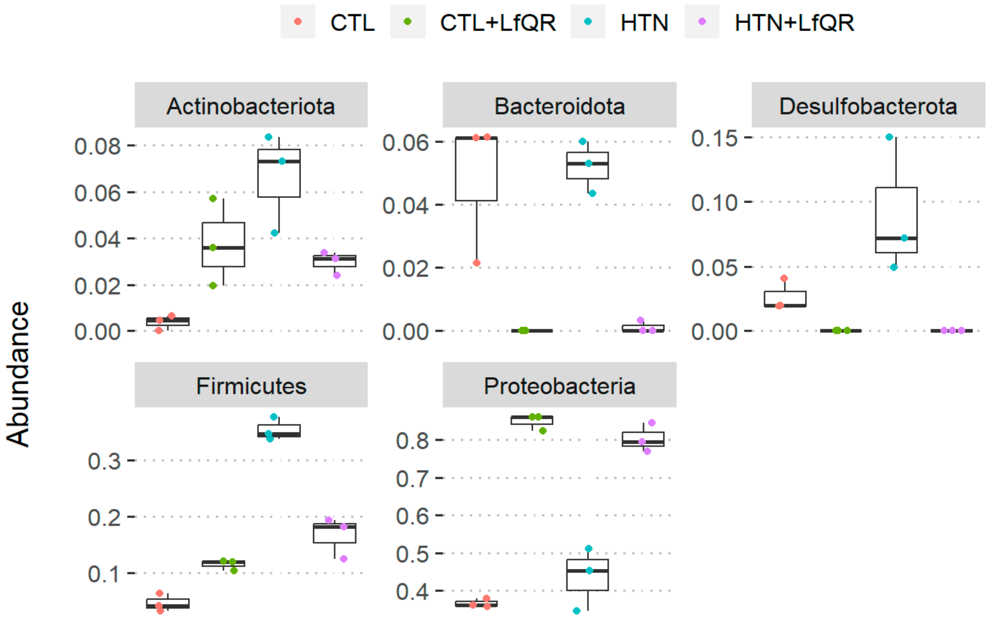

Supplementary Figure S2. Relative abundance of microbial phyla *in vitro* colonic fermentation.

HTN: Hypertension fermentation medium. HTN-LfQR: Hypertensive fermentation medium with LfQR. CTL: Control fermentation medium; CTL+ LfQR: control fermentation medium with LfQR. LfQR: Nutraceutical formula with *L. fermentum*, quercetin and resveratrol

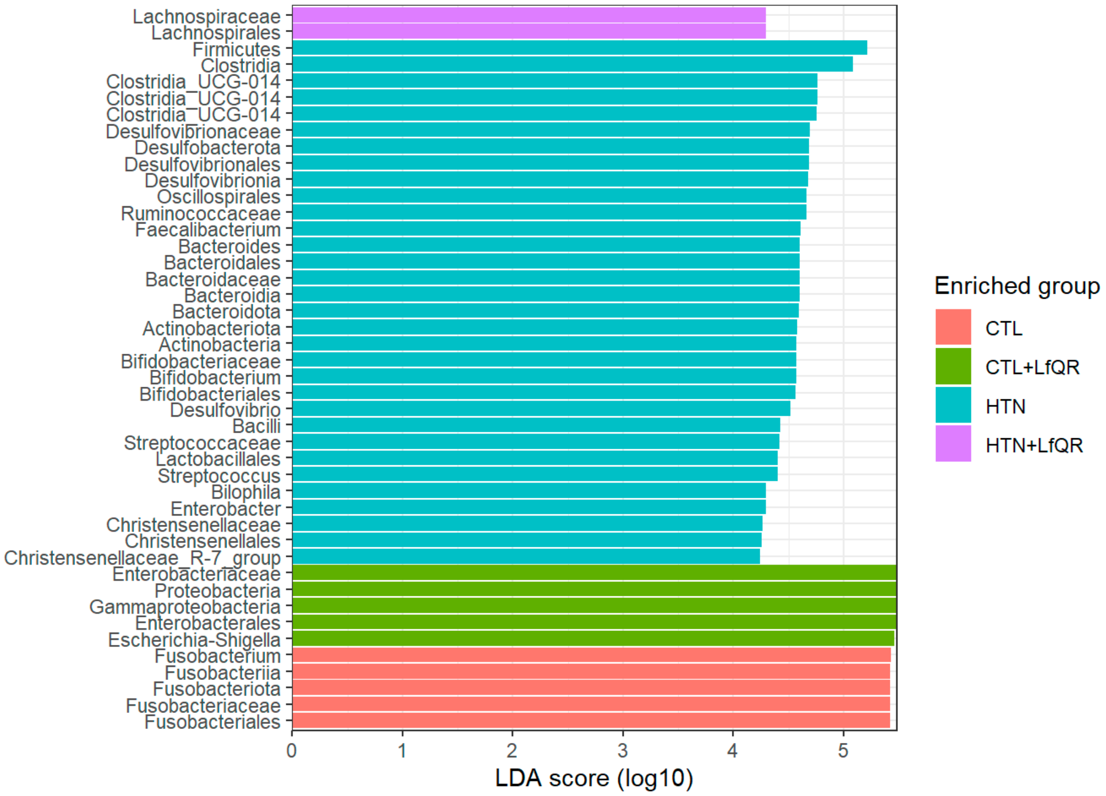

Supplementary Figure S3. LDA analysis of microbial genera *in vitro* colonic fermentation.

HTN: Hypertension fermentation medium. HTN-LfQR: Hypertensive fermentation medium with LfQR. CTL: Control fermentation medium; CTL+ LfQR: control fermentation medium with LfQR. LfQR: Nutraceutical formula with *L. fermentum*, quercetin and resveratrol
